# Quality Evaluation for Colored Point Clouds Produced by Autonomous Vehicle Sensor Fusion Systems

**DOI:** 10.3390/s25041111

**Published:** 2025-02-12

**Authors:** Colin Schaefer, Zeid Kootbally, Vinh Nguyen

**Affiliations:** 1 Department of Mechanical and Aerospace Engineering, Michigan Technological University, 1400 Townsend Drive, Houghton, MI 49931, USA; colinsch@mtu.edu; 2 Intelligent Systems Division, National Institute of Standards and Technology, 100 Bureau Drive, Gaithersburg, MD 20899, USA; zeid.kootbally@nist.gov

**Keywords:** LiDAR, camera, sensor fusion, colored point clouds, automated driving systems, metrology

## Abstract

Perception systems for autonomous vehicles (AVs) require various types of sensors, including light detection and ranging (LiDAR) and cameras, to ensure their robustness in driving scenarios and weather conditions. The data from these sensors are fused together to generate maps of the surrounding environment and provide information for the detection and tracking of objects. Hence, evaluation methods are necessary to compare existing and future sensor systems through quantifiable measurements given the wide range of sensor models and design choices. This paper presents an evaluation method to compare colored point clouds, a common fused data type, among two LiDAR–camera fusion systems and a stereo camera setup. The evaluation approach uses a test artifact measured by the fusion system’s colored point cloud through the spread, area coverage, and color difference of the colored points within the computed space. The test results showed the evaluation approach was able to rank the sensor fusion systems based on its metrics and complement the experimental observations. The proposed evaluation methodology is, therefore, suitable towards the comparison of generated colored point clouds by sensor fusion systems.

## 1. Introduction

One of the core aspects that differentiate autonomous vehicles (AVs) from manually driven vehicles is their perception system. As AV development continues, it has become apparent that their perception system will need to be composed of multiple and distinct types of sensors to ensure AVs can perceive their surroundings for all possible scenarios and weather events [1,2]. Such a sensor fusion system needs to gather data from the surrounding environment for complex decision-making [3]. There are numerous approaches to a perception fusion system for an AV [4] due to the plethora of design choices, including what types of perception sensors to use, how many to use, and their placement on the AV. Hence, there is a need to evaluate the performance of all these unique perception systems under standardized methods. Currently, no standardized test methods exist for evaluating AV sensor fusion systems.

Light detection and ranging (LiDAR) sensors and cameras are commonly used sensor fusion systems in AVs [5,6]. However, LiDAR and cameras generate different data, resulting in difficulties of individually quantifying and comparing perception performance. In addition, LiDAR–camera sensor fusion architecture can vary from system to system [5,6]. However, they all share the primary purpose of classifying objects in the environment, whether for mapping or detection [7,8]. Hence, the goal of a LiDAR–camera fusion perception system is to provide data on the driving environment for automated driving functionality. Therefore, a standard evaluation method would need to test an AV’s LiDAR–camera sensor fusion ability to accurately observe the environment and address the nature of perception observations.

One fused data type in LiDAR–camera sensor fusion is colored point clouds, which are used as a representation of objects and for capturing differences in appearance. Colored point clouds are the closest data type to how humans see their surroundings because they contain color and distance values of an environment. While test artifacts have been developed for camera images [9] and stereo camera setups [10], the assessment of colored point clouds is still an active area of study. Point Cloud Quality Assessment (PCQA) is an evaluation technique to measure the distortion an object within a point cloud. PCQA can be implemented through a subjective or objective approach. Subjective PCQA involves using human subjects to grade point cloud objects based on their discretion, while objective PCQA uses computations and calculated metrics to compare point clouds of the same object [11]. Objective PCQA is further categorized by the presences of a reference model of the object. Objective PCQA techniques can either compare a distorted model to a highly dense reference model and measure the differences [12,13], use some features of a reference model for comparison [14,15], or use no reference model and develop a different method to measure the distortion [16,17]. These techniques are known as full-reference, reduced-reference, and no-reference methods, respectively. There also exist prior methods for full-reference PCQA using public datasets [12,13]. For instance, test objects have been integrated into public datasets for the evaluation of point clouds [18,19]. However, such an approach is expensive and no standard exists to ensure the point cloud data are consistently accurate between research groups. Though no-reference methods have been proposed, most of the methods used machine learning techniques [20,21], which can be computationally expensive and subjective to the training data. Recent methods also involve injecting features within point clouds [16] or using private point cloud data to prove validity [17]. Unfortunately, most PCQA methods evaluate the distortion in point clouds using the entire point cloud representation of an object. Nevertheless, objective PCQA is seen as more suited for the evaluation of point clouds because subjective PCQA has been shown to lead to variable scores based on the subject’s preference [22].

The flaws of subjective PCQA can be alleviated through applying standardized image quality assessment test methods [23,24]. For instance, camera standards can be leveraged to consistently determine image quality by providing observers with three images at a time to evaluate their quality with characteristics including color difference and sharpness [25]. In addition, LiDAR standards have been implicitly covered in test methods and documentation guidelines for evaluating perception stacks of automated driving capabilities [26]. While these standards include detectability, which is inferred from spread and area coverage, these test methods do not specifically quantify LiDAR. Furthermore, these standard test methods do not apply to colored point clouds.

**Figure 1:**
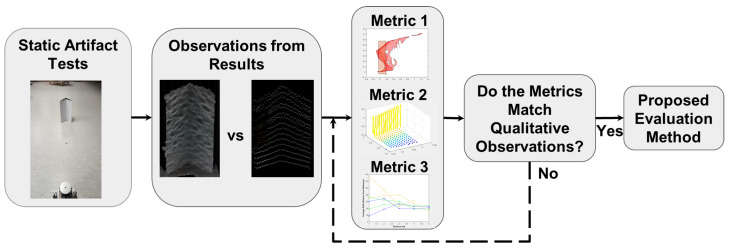
The overview of the development of the colored point cloud evaluation method. The research approach starts with performing the tests to obtain raw data consisting of colored point clouds representations of the test artifact.

The research presented in this paper aims to fill the gap in the literature with respect to a repeatable method to evaluate colored point cloud data from different AV sensor fusion perception systems using a novel application of metrics (spread, area coverage, and color difference) and test artifacts. The proposed evaluation method uses a test artifact developed by the authors to evaluate multiple aspects of sensor fusion systems. The research approach in this paper is as follows: First, data were acquired by performing a scan test at varying distances from the different sensor fusion systems simultaneously. The resulting data were reviewed to find clear distinctions between the colored point clouds from the different fusion systems. These observations served as benchmarks for the evaluation method to recreate using quantitative metrics. The calculations and the metrics used within the proposed method were refined until they produced desirable results. Figure 1 shows an overview of the research approach used to develop the proposed metrics. Note that the main goal of this research is the evaluation process and not to determine which of the presented sensor fusion systems is best for an AV application.

## 2. Methodology

This section provides a description of the general experimental setup. First, the setup for test artifact scan tests is described along with the sensor hardware and the sensor fusion. Afterwards, the metrics used within the proposed evaluation method are presented.

### 2.1. Experimental Setup

For these static object tests, a Velodyne VLP-16 Puck (Velodyne, San Jose, CA, USA) [27] was used for the LiDAR, and an Intel D435i Realsense Depth Camera (Intel, Santa Clara, CA, USA [28] was used to obtain two-dimensional (2D) images of the test environment for the LiDAR–camera fusion systems. The Realsense camera produces a colored point cloud created by its internal stereo camera system in addition to the 2D image output. To obtain the raw data from these sensors, the respective open-source Robotic Operating System (ROS) drivers for each of the sensors were used. For data collection, the ROS drivers ran simultaneously, and the sensors were fixed to a mount that placed the LiDAR 0.114 m above the camera. To develop the proposed evaluation method, three separate sensor fusion systems were used to compare their colored point cloud representation of the test artifact. These three fusion systems were the Matrix Laboratory from MATLAB 2023a (MathWorks, Natick, MA, USA) fusion function, a publicly available sensor fusion ROS driver (lidar-camera-fusion 11.1.1, University of Alicante, Alicante, Spain) [29,30], and the output of the stereo camera setup within the Realsense camera. The MATLAB function used was the “fuseCameraToLidar()” function, which requires a point cloud, a 2D image, the camera’s intrinsic parameters, and the extrinsic transformation matrix between the LiDAR and camera. These same arguments were needed for the LiDAR–camera fusion ROS drivers. The difference between these fusion techniques is the MATLAB fusion function was performed after the data were collected as compared to the live data fusion from the ROS drivers. In addition, the MATLAB fusion function only overlays the image onto the point cloud giving color values to each point, while the ROS drivers interpolates new points between the LiDAR’s laser scan layers in addition to giving color values [29,30]. The extrinsic transformation matrix between the LiDAR and camera was calibrated using the Lidar-Camera Calibration MATLAB 2023a app. While these two sensor fusion methods used a 3D point cloud and a 2D image, the Realsense stereo camera setup uses two identical cameras to measure depth. An optional infrared projector exists within the Realsense unit that can improve the stereo camera’s depth accuracy, but this light source was not used during testing. In this work, each sensor fusion system is given a short-hand reference: the MATLAB 2023a fusion function, ROS drivers, and the Realsense stereo camera are referred to as sensor fusions “Without Interpolation”, “With Interpolation”, and using a “Stereoscopic” design, respectively. (Certain equipment, instruments, software, or materials are identified in this paper in order to specify the experimental procedure adequately. Such identification is not intended to imply recommendation or endorsement of any product or service by the National Institute of Standards and Technology, nor is it intended to imply that the materials or equipment identified are necessarily the best available for the purpose).

**Figure 2:**
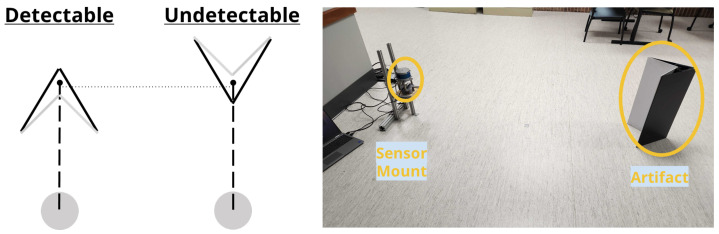
A schematic showing a top view of the test setup for each artifact side with the sensor shown in grey (**left**). An example of a detectable test at 2 m with the artifact and sensor mount circled in yellow (**right**).

The testing for data collection involved a static artifact placed a distance away from the sensor mount. The artifact used for testing consisted of two sides: a detectable and undetectable side, which were composed of two plates to be either “detectable” or “undetectable” to a LiDAR when the corresponding side is in view. Hence, the perception sensor’s ability to detect the artifact is hampered or enhanced by the physical design of the side of the artifact. For the detectable side, the artifact exhibits a white color for higher reflection intensity for LiDARs, a rough texture to ensure light is diffused equally in all directions, and a concave shape to direct the reflected light back towards its source. The undetectable side has a black color for low reflection intensity for LiDARs, a smooth surface finish to better reflect the emitted light in one direction, and a convex shape to ensure the reflected light never returns to the sensors. These two distinct sides can be used to evaluate a sensor fusion system between LiDARs and cameras to evaluate their perception capabilities under ideal and difficult conditions. Using a single artifact ensures ease of manufacturing and simple implementation for a test to evaluate the sensor fusion systems under the same conditions. The detectable side flat plates had dimensions of 0.61 m by 0.21 m arranged in a 45° 0.01 cm concave shape towards the sensors. The undetectable side had 0.61 m by 0.30 m plates arranged in a 60° 0.01 cm convex shape towards the perception sensors. Colored point cloud data were collected for both sides at 1 m, 2 m, 3 m, 4 m, and 5 m away from the sensor mount’s center. Figure 2 provides a visual display of the endpoints of these distances and an example of one of these scan tests. All the tests presented were performed in one location: a general lab space with dimensions of approximately 10.72 m × 8.99 m × 2.74 m. Data shown in this work were collected at night with the overhead lights in the lab space acting as the only source of visible light.

### 2.2. Evaluation Method and Metrics

The calculations performed within the proposed evaluation method are discussed in this section. First, an explanation of how the points representing the artifact were separated from the rest of the environment is presented. This section discusses the process of splitting the artifact point cloud into a left and right side for ease of analysis and obtaining their reference planes. The point cloud coverage analysis is then introduced. Next, the spread of the point cloud around the expected location of the test artifact’s side is discussed. Finally, the color difference between the artifact points and the reference or expected color of the artifact is presented.

#### 2.2.1. Preliminary Evaluation Steps

The points representing the test artifact must first be isolated and stored in a separate dataset from the rest of the environment. After data collection, the artifact point cloud was split into two separate sets of points corresponding to the left side and the right side. From these two point cloud sets, a reference plane was created to estimate where the two halves of the visible side of the test artifact are located within the colored point cloud.

**Figure 3:**
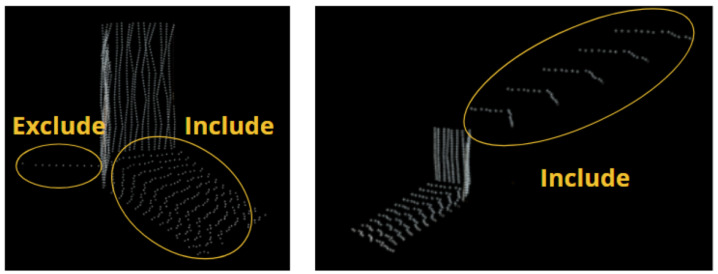
Images of sample results visually describing the conventions followed when isolating the points of the test artifact within the interpolation fusion data.

**Figure 4:**
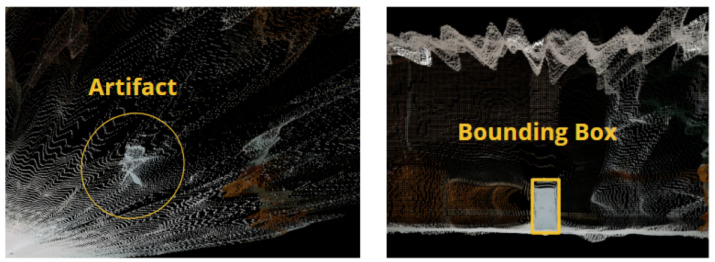
Results from a sample test showing an example of how a bounding box was used to isolate the test artifacts points in stereoscopic data for tests at distances greater than 3 m.

The artifact points within the data from the fusion system without interpolation were isolated from the rest of the environment. The following convention was used when isolating the artifact points in the fused data with interpolation fusion: points that were above the ground and were between the sensors and the artifact were included in the artifact set, while single-line trailing points emitting from the back of the artifact were excluded. Figure 3 provides visual examples of this convention. The 1 m and 2 m scan tests of the stereoscopic data produced clear artifact point sets with the only encountered obstacle separating the ground points from the artifact ones. The remaining tests did not contain a distinguishable artifact. These issues were expected because the maximum range listed within the Realsense camera’s manual is 3 m. For these tests, the artifact sets were cropped by viewing the full point cloud from the camera’s position and creating a 2D bounding box covering the estimated location of the visible artifact’s side. A visual demonstration of this process is shown in Figure 4. These cropped point clouds were analyzed, and their performance was compared to the other fusion system for completeness.

Before continuing with the evaluation, the ground plane for each sensor fusion system was established for future calculations. A single, random point cloud result was taken from the test set for each of the fusion systems. Within these point clouds, the ground points were isolated from the rest of the environment. The ground plane was estimated from these points and its parameters were stored for evaluation.

**Figure 5:**
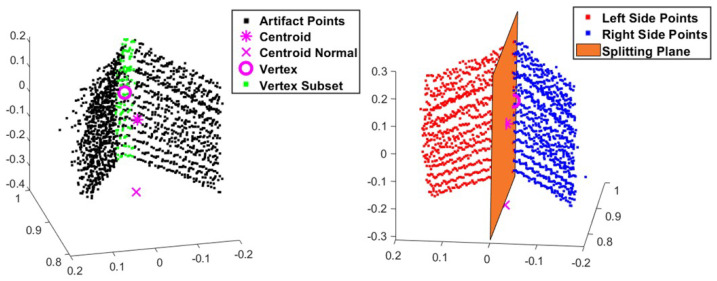
Developing the splitting plane to classify the artifact points based on their location. Coordinates are in meters.

Once the artifact point cloud sets were established, each set needed to be split into two to represent the left and right plates that compose one of the test artifact’s sides. A splitting plane formation process was created to ensure consistency throughout the analysis. Three points were calculated from the artifact point cloud to define this plane: the centroid of the artifact points was determined by calculating the average location value, the normal point which is a point that is in line with the centroid point calculated by using a directional vector equal ground plane’s normal vector and is located near the ground plane, and the centroid point (referred to as the vertex point) of a bounding box encompassing the vertex of the artifact. The splitting plane and its point-normal equation were derived from these three points. Once the plane was formed, the coordinates for each artifact point were inserted into the splitting plane’s point-normal equation. The sign of the result would indicate on which side of the splitting plane the points were located. Figure 5 illustrates this splitting process.

**Figure 6:**
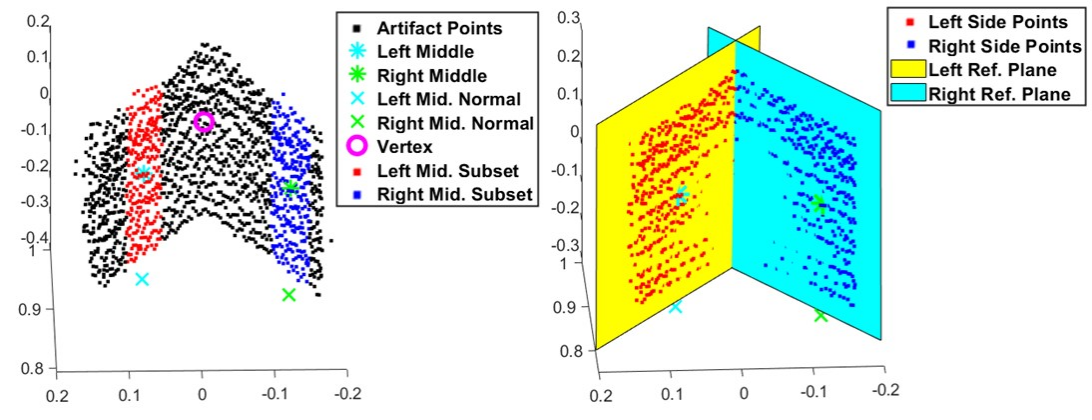
The process of creating the reference planes for both sides of the test artifact points. Coordinates are in meters.

With the two halves of the visible artifact’s side separated, the reference plane for each was determined. Similar to creating the splitting plane, three points were found to form each reference plane. To ensure that the reference planes intersected at their vertex, both reference planes shared the vertex of the splitting plane. The second point was calculated from finding the average location of a subset of points in the center of the two sides. The final point was calculated by using this middle point and the normal vector of the ground plane to find a point that lies near the ground plane. These points controlled the angle between their corresponding reference plane and the other reference plane. Also, these points ensured that the reference planes were normal to the ground plane. With these two perpendicular lines and the shared vertex point, the reference planes and their point-normal equations were derived and used in the point coverage and spread metrics. Figure 6 demonstrates the reference plane creation process. The process for determining this middle point worked well with clear artifact point clouds. However, the process was difficult to implement with the noisy stereoscopic data. Through the rigorous iteration of the subset limits for the middle points, usable reference planes were produced for the noisy artifact point clouds.

#### 2.2.2. Point Coverage

With the reference planes, the artifact points of both sides can be projected onto their respective reference plane to obtain a 2D representation of the artifact point cloud. The following were used to calculate the coordinates of each point relative to the reference plane’s coordinate system ($p_{x}^{R}$ and $p_{y}^{R}$) in their corresponding point cloud reference frame:

(1) $$p_{x}^{R}=R_{X}\cdot \left(p_{xyz}-R_{o}\right)$$

(2) $$p_{y}^{R}=R_{Y}\cdot \left(p_{xyz}-R_{o}\right)$$

where $R_{X}$ represents the *x*-axis of the reference plane, $R_{Y}$ is the reference plane’s *y*-axis, which is equal to the ground plane’s normal vector, $R_{o}$ is the origin point of the reference plane’s coordinate system, and $p_{xyz}$ is an artifact point’s location relative to the full point cloud. Note that $R_{X}$ was determined by finding the vector that was perpendicular to its respective reference plane’s *y*-axis vector and normal vector (*z*-axis). The middle normal point used in creating the reference plane acted as the origin for its corresponding reference plane because the point was always located below the artifact points.

**Figure 7:**
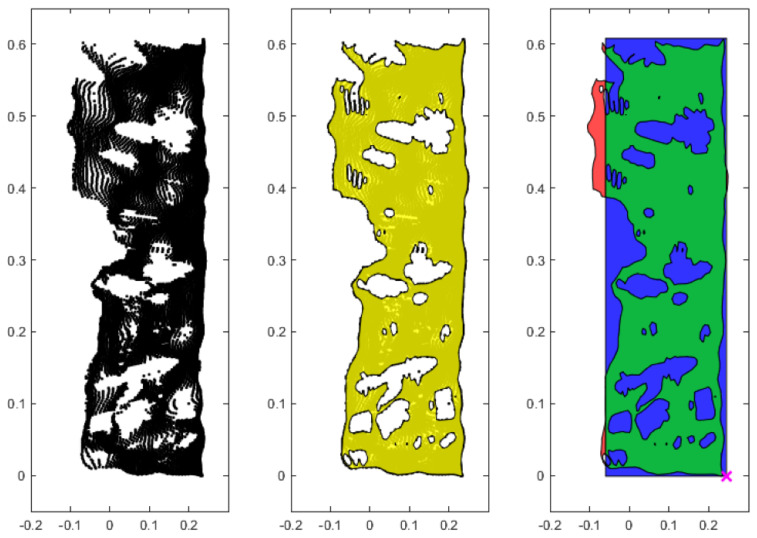
A series of plots visually describing the sequence of determining the point coverage of a set of artifact points. For these images, the stereoscopic data of the left−undetectable side at 1 m test was used, with the projected artifact point on the left reference plane shown in black (**left**), forming the shape of the total point coverage shown in yellow (**middle**), and determining the point coverage of the test artifact’s expected area with the artifact’s bounding box in blue, the centroid normal in pink x, point coverage in green, and error in the point coverage in red (**right**).

The artifact points within the reference planes’ coordinate systems allowed for easier analysis of these points to investigate how well they covered the expected area of the test artifact. Finding the point coverage area that was within the expected test artifact area relative to the reference planes determined the quality of the point cloud coverage for each test. The total point coverage of the point cloud relative to their respective reference plane was first found. A neighborhood set for each artifact point was determined by using the Radius Nearest Neighbors method with increasing radius values of 0.01 m, 0.03 m, 0.05 m, 0.07 m, and 0.10 m corresponding to the data with test distances of 1 m, 2 m, 3 m, 4 m, and 5 m. With each neighborhood set, a boundary was placed around the points using the “boundary()” MATLAB function. A shrink value of 0.01 was implemented to ensure the neighborhood sets had convex shapes, to cover the most area, and to output a cleaner appearance. With these boundary sets, a polygon (“polyshape()” object in MATLAB) could be created by using the boundary points as its vertices. Removing the overlapping area between the entire set of polygons results in the total point coverage area of the artifact points with respect to the reference planes. An expression for the total point cloud coverage of the artifact set ($P_{\Sigma }$) is given in the following:

(3) $$P_{\Sigma }=N_{1,A}\cup N_{2,A}\cup \ldots \cup N_{n,A}$$

where $N_{A}$ denotes the area of a neighborhood set of one of the artifact points and *n* is the number of points within the artifact set. Creating the expected artifact area in the reference planes was performed as follows. First, the bottom left or right corner of the artifact’s side was projected onto the reference plane. This point was determined by first finding intersection lines between the two reference planes and both intersection lines between the reference planes and the ground plane. With these three lines, their intersection point was calculated and projected onto the reference planes as the bottom corner of the visible test artifact’s sides. Using the physical dimensions of the test artifact, the area of each half was overlaid on the reference planes with the projected artifact points’ total point coverage shape. The intersection between these two shapes provides the point coverage of the expected area for the artifact points, while the leftover area of the total coverage shape represents the error or the incorrect point coverage that lies outside the expected area. The following equation represents the point coverage of the expected area (*P*):

(4) $$P=P_{\Sigma }\cap R_{A}$$

where $R_{A}$ represents the estimated area of coverage of the artifact in the reference plane. Figure 7 provides a sequence of plots visually describing the process of obtaining the point coverage of the projected points. The point coverage of the expected area expressed in a percentage value ($P_{\%}$) was found by dividing the intersection area by the surface area of the test artifact’s side, as shown in the following equation:

(5) $$P_{\%}=\frac{P}{R_{A}}$$

The other percentage term is the point coverage error ($P_{\epsilon }$) calculated as:

(6) $$P_{\epsilon }=\frac{P}{P_{\Sigma }}$$

#### 2.2.3. Spread

The equation shown below was used to calculate the shortest distance ($p_{d}$) between an artifact point and the reference plane.

(7) $$p_{d}=R_{n}\cdot \left(p_{xyz}-R_{o}\right)$$

where $R_{n}$ represents the normal vector of the reference plane. These distance values help describe the spread of the artifact points relative to one of their corresponding reference planes. Once this distance value was determined for each point, the Root-Mean-Square (RMS) variable was calculated for each half.

#### 2.2.4. Color

The color metric in this paper evaluates the red–green–blue (RGB) value of the points disregarding the reference planes of the artifact. The RGB value of each point is compared to a reference RGB value (*r*) using the color difference (*CR*) variable as follows:

(8) $$p_{CR}=\sqrt{{\left(p_{R}-r_{R}\right)}^{2}+{\left(p_{G}-r_{G}\right)}^{2}+{\left(p_{B}-r_{B}\right)}^{2}}$$

where $p_{R}$, $r_{R}$, $p_{G}$, $r_{G}$, $p_{B}$, and $r_{B}$ are the measured colors and reference colors, respectively, on the red, green, and blue scales. An average for the full artifact set was then calculated. Reference RGB values of (146, 145, 143) and (189, 188, 186) were visually determined for the left and right detectable sides. These were calculated by finding the average RGB value of 10 randomly selected pixels from an image of the test setup. It was assumed the undetectable side was pure black color, which corresponds to a reference value of (0, 0, 0) for both sides.

## 3. Results

**Figure 8:**
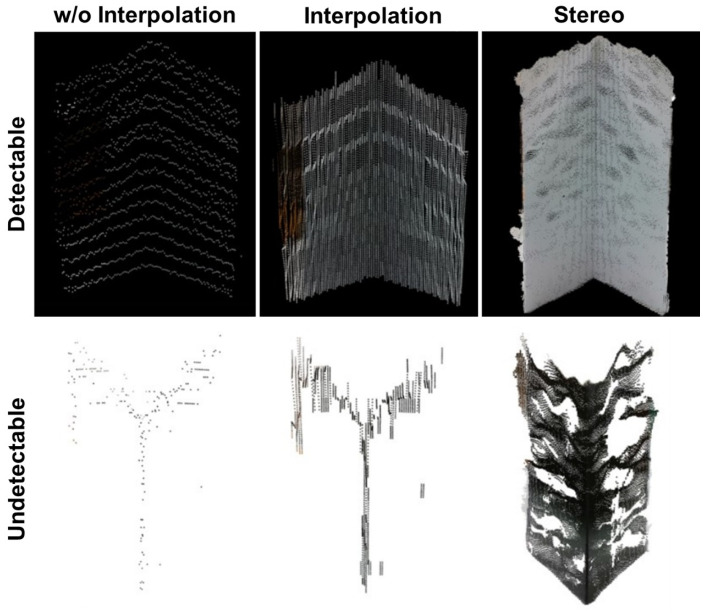
The raw point cloud of each testing configuration at the test distance of 1 m.

The results for each of the evaluation aspects are presented and discussed to determine if the quantitative results follow the reported observations. For each metric, the observations are discussed first followed by the results produced by the evaluation method. The corresponding analysis for the point cloud coverage, spread, and the color difference of the points are discussed. Figure 8 provides exemplary data of the point clouds.

### 3.1. Point Cloud Coverage

To summarize the observations relating to point cloud coverage, coverage was the best at close distances and at tests with the detectable side. Generally, as distance increased, the greater the error and the less the coverage of the expected area. Also, for most of the tests, there was a greater amount of points for the detectable tests as compared with the undetectable side. With fewer points, the undetectable side had less coverage than the detectable tests. The stereoscopic data consistently had the most coverage because they had the largest amount of points, while the interpolation fusion system had the next best amount of coverage. As expected, the fusion data without interpolation had the least coverage area because they always had the least amount of points. These relationships can clearly seen within Figure 8.

**Figure 9:**
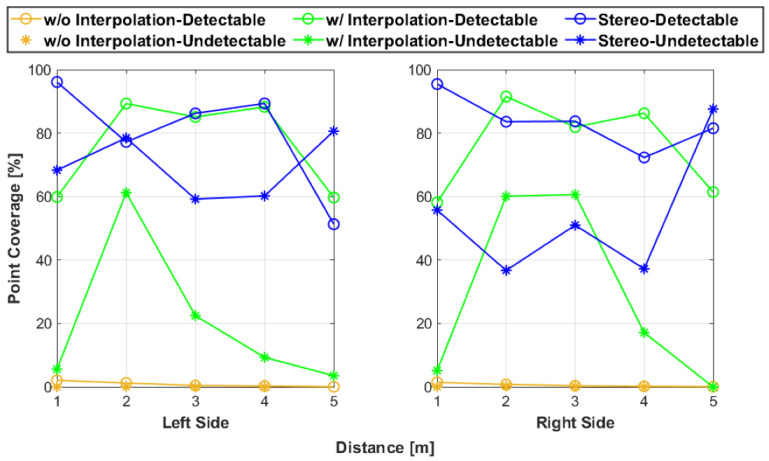
The point coverage results of the test represented as the percentage of the artifact’s expected area the point cloud covers within its enclosed area.

**Figure 10:**
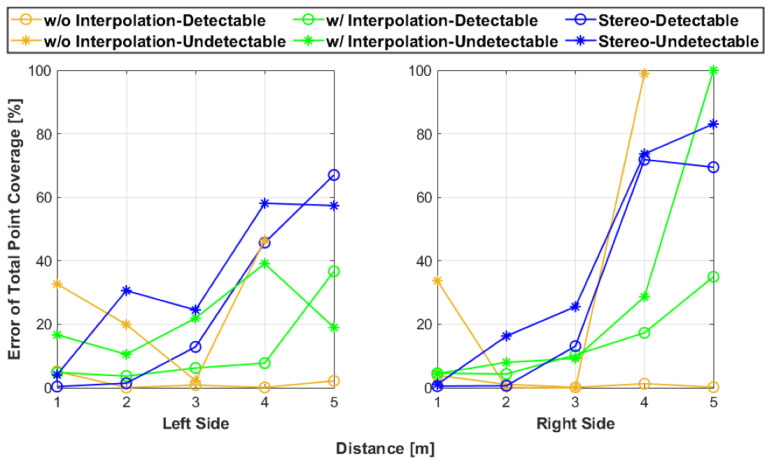
The error in point coverage for each test represented as the percentage of the total point coverage located outside the expected area region of the test artifact.

Figure 9 provides the results for point coverage of the expected area while Figure 10 contains the error in coverage plots. The point coverage results are similar to the observations: the stereoscopic data with the detectable side at 1 m were the best overall, without interpolation, fusion points covered very little of the area, and all configurations generally decreased with increasing testing distance. In addition, for most of the tests, the undetectable side performed worse than the detectable side tests. The increase in point coverage at 2 m for undetectable was expected because the LiDAR sensors performed best at that test distance when scanning the artifact’s undetectable side. This phenomenon was encountered during the development of the artifact’s design. Investigating the point coverage error plots also generally followed the prior observations. As the test distance increased, the coverage error increased for all tests. The errors for undetectable side tests were mostly greater than their counterparts. Also, the error results illustrate that despite having little point coverage, the fusion without interpolation for the detectable side was relatively error-free. An unanticipated behavior within the evaluation results was that some of the right side error values were far larger than the left side values. This difference is most likely caused by the subjective nature of the artifact isolation process and the imperfect estimation of determining where the artifact’s expected area region lies relative to the artifact’s point set.

### 3.2. Spread

The artifact points closely clustered around the reference planes for 1 m and 2 m for all fusion techniques. However, as the test distance increased, the distance between the artifact points and the reference planes increased, with some points reaching almost a meter from where the test artifact’s expected location. This increase of spread was only present in the interpolation and stereoscopic data, while the artifact points from the fusion without interpolation were consistently clustered near the reference planes. Figure 8 provides little insight in this point spread behavior. However, Figure 3 and Figure 4 imply how the point spread increased with the test distance because the point clouds presented in both figures were included in the full dataset for the evaluation method.

**Figure 11:**
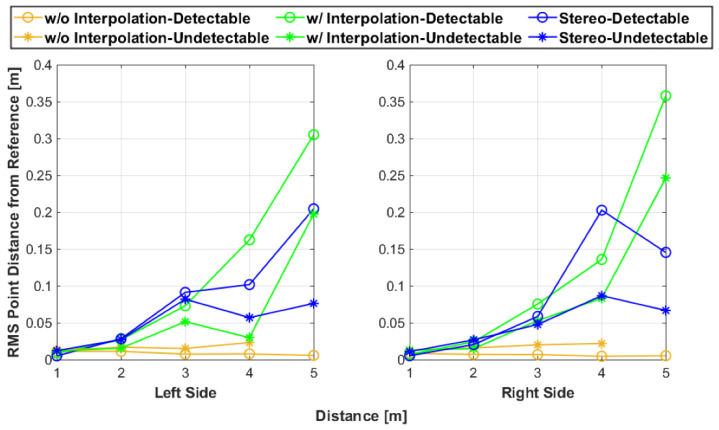
The RMS value of the distance between the artifact points and their respective reference plane.

The results shown in Figure 11 match the general observations: the without interpolation fusion points consistently ranked the highest with the shortest RMS values as compared to the other fusion methods. As with the other two fusion methods, as the test distance increased, the point spread increased their artifact sets. Interestingly, the detectable side ranked the lowest as compared to their undetectable counterpart. This behavior could be attributed to subjective error from the isolation process of the artifact points. It was easier to isolate the light-colored detectable side points than the dark-colored points of the undetectable side because of the better visibility. Also, as seen during the development of the test artifact, there were fewer points in the undetectable artifact point clouds as compared to the detectable side point clouds. The points further away from the reference planes were most likely not registered in the raw-colored point clouds because of their low-intensity backscattering values.

**Figure 12:**
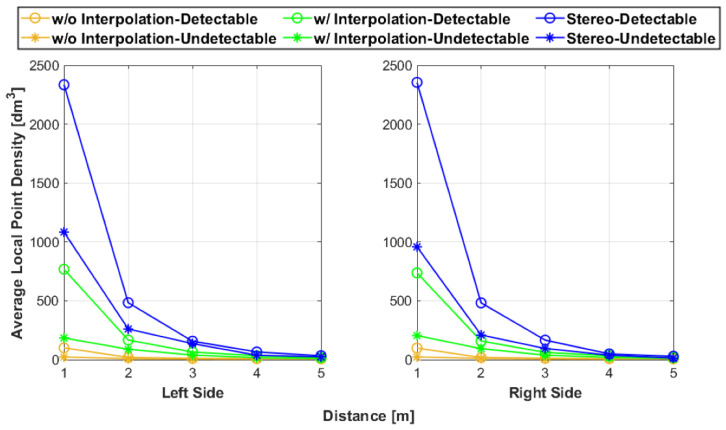
The average LPD of the artifact point clouds.

In addition, a comparative analysis was conducted with prior literature metrics for point cloud analysis. Specifically, the local point density (LPD) was computed [31]. The LPD refers to the number of points surrounding a query point in a given volume. Hence, the LPD was calculated for every point in the point cloud and then the average of the LPD values was determined. The radius of the spherical volume surrounding each point was 0.05 m. Figure 12 shows the results of the average LPD values. As expected, as the distances from the artifact increased, the LPD decreased. However, unlike the RMS values, the LPD is intrinsic and does not reference an artifact. Hence, the results in Figure 11 are more intuitive towards determining accuracy than the LPD since it quantifies the spacing of the point cloud to the reference rather than the spacing between points within the point cloud.

### 3.3. Color

The main trends noticed were that both the fusion systems with and without interpolation had significant color error close to the sensors as shown in Figure 8, but this error decreased as the distance increased. These inaccuracies were most likely caused by the calibration error within the LiDAR and camera fusion system. The stereoscopic data generally performed better in representing the artifact’s color because of the use of a stereo camera configuration and the process of isolating the artifact points benefited the system greatly. Within Figure 8, the stereoscopic data had little color variation as compared to the other fusion systems.

**Figure 13:**
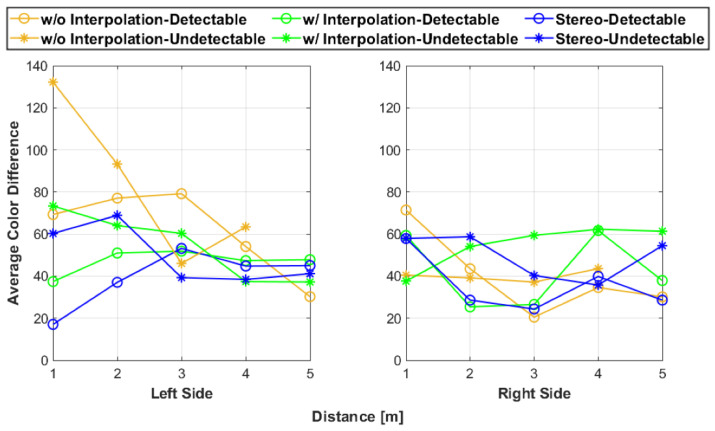
The average color difference values for each test.

Figure 13 matches with some of the expected behavior found in the observations. Both the fusion with and without interpolation performed worse at close distances than the stereoscopic data for the left side, but as the test distances increased, all the data seemed to converge to the same range of values for both the left and right sides. This behavior is valid because as the distance increased, the color within the pixels had less variation since the test artifact was smaller relative to the camera. Despite capturing the LiDAR–camera calibration error, the color difference results did not capture all the observations related to the color quality of the point clouds.

**Figure 14:**
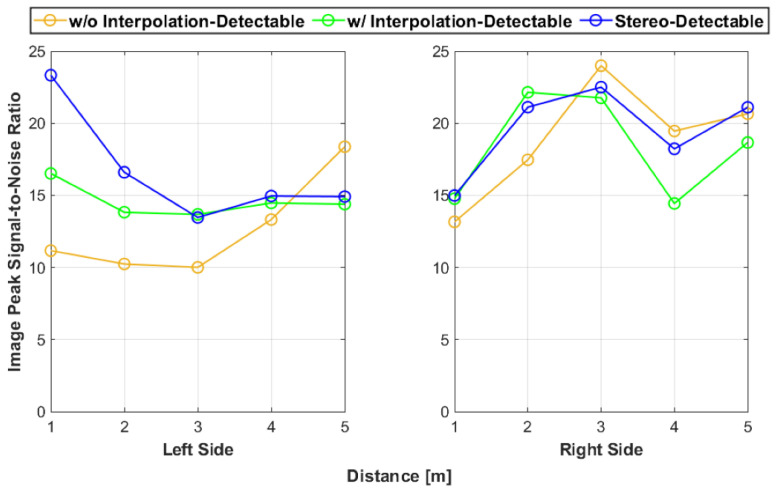
The PSNR image values for each test. Note that the PSNR could not be calculated for the undetectable side since the reference color was (0, 0, 0).

In addition, a comparative analysis of the average color distance with the traditional peak signal-to-noise ratio (PSNR) for image quality was conducted [32]. The PSNR is expressed as the ratio between the maximum possible value (power) of the reference image and the power of corrupting noise, which is expressed as the mean square error in the color difference. Hence, the PSNR is expressed as the following equation:

(9) $$PSNR=20\times \log_{10}\left(\frac{{MAX}_{I}}{\sqrt{MSE}}\right)$$

where MSE is the mean squared error of the color difference and $MAX_{I}$ is the max intensity of the reference value. Figure 14 shows the results of the PSNR analysis. As expected, Figure 14 exhibits the inverse behavior of Figure 13. This is because the noise increased as the color difference increased, which resulted in a lower PSNR value. However, note that the theoretical peak image value of the PSNR for the undetectable side was (0, 0, 0). Therefore, the PSNR could not be calculated for the undetectable side. Hence, the color difference metric seems more sufficient for testing with the reference artifact used in this work.

To summarize the results, the stereoscopic fusion system had the best performance of all the results at 1 m with the detectable side. However, the error and spread of the points greatly increased with the test distance. The fusion system without interpolation had the lowest point coverage and generally had the most color variation, but the error in the point coverage and its spread were small as compared to the other two fusion systems. The fusion system with interpolation had the least extreme results since it did not have the worst or best values for each evaluation metric, regardless of the test parameters. Note that the methods in this work are interlinked with the proposed test artifact. Automated driving environments encounter a large variety of obstacles including pedestrians, signage, and foliage. To apply this research towards objects detected in the driving environment, this work would have to be either adapted or extrapolated to such objects. With respect to adapting this work for objects in the driving environment, a representative 3D model of the objects should be constructed. Equations (1)–(8) can then be used based on the 3D model as a reference to compute the point cloud coverage, the spread, and the color difference. Note that to compute the color difference, the 3D model should also have reference color values that can be acquired using a colorimeter. Alternatively, another approach would be to conduct these tests using this test artifact, and then extrapolate the results to a driving environment object. For instance, the differences in the geometry and color between the test artifact in this work and the driving environment object would be identified and examined to approximate the LiDAR–camera sensor fusion performance for the driving environment object.

## 4. Conclusions and Future Work

The proposed evaluation method in this work can be applied to colored point clouds produced by AV sensor fusion perception systems. The results from this method lead to similar conclusions as observations of the colored point cloud data. Due to the simplicity of the evaluation metrics, they are effective in analyzing the colored point clouds and provide quantifiable measurements to determine the strengths and weaknesses in the perception accuracy of sensor fusion systems. Point cloud coverage metrics distinguish which colored point cloud accurately represents the artifact shape in the point cloud space. The spread value provides an insight into the spacing of points relative to the test artifact’s location compared to the LPD, which intrinsically determines a point cloud’s spacing. Finally, averaging the color difference value of the point cloud helps determine whether the 2D image pixels are calibrated to the artifact, while the PSNR is insufficient for reference artifacts that are a black color. The next steps for this evaluation method are to improve the processes implemented, especially refining the preliminary steps to ensure consistency between the sensor fusion systems. These steps are the most subjective and are prone to cause errors within the analysis. In addition, developing a more sophisticated approach, such as with machine learning, to evaluate the point’s color can be conducted because this metric is the weakest of the three in capturing the accuracy of the point cloud. Also, future work may involve testing other sensor fusion systems using this method to ensure consistency in results and confirm that the method is applicable to other sensors.

## Data Availability

Data are contained within the article.
